# Growth Dynamics and Diversity of Yeasts during Spontaneous Plum Mash Fermentation of Different Varieties

**DOI:** 10.3390/foods9081054

**Published:** 2020-08-04

**Authors:** Magdalena Skotniczny, Paweł Satora, Katarzyna Pańczyszyn, Monika Cioch-Skoneczny

**Affiliations:** Department of Fermentation Technology and Microbiology, University of Agriculture in Krakow, Balicka 122, 30-149 Krakow, Poland; pawel.satora@urk.edu.pl (P.S.); katarzyna.panczyszyn@urk.edu.pl (K.P.); monika.cioch@urk.edu.pl (M.C.-S.)

**Keywords:** 26S rRNA gene, D1/D2 domains, plum brandy, slivovitz, yeast ecology

## Abstract

The influence of fruit varieties on yeast ecology during spontaneous plum mash fermentation was investigated. Yeast colonies were isolated from mashes obtained from four plum varieties throughout fermentation in laboratory conditions during two consecutive years. The yeast strains were differentiated by random amplification of polymorphic DNA (RAPD-PCR) and identified by the 26S rDNA D1/D2 sequence analysis. *Hanseniaspora uvarum*, *Metschnikowia* spp. and *Pichia kudriavzevii* were the dominant yeasts during the early stages of plum mash fermentation, while the middle and end phases were dominated by *Saccharomyces cerevisiae*. The strains of *Candida sake*, *Nakazawaea ernobii*, *Pichia kluyveri*, *Rhodotorula mucilaginosa* and *Wickerhamomyces anomalus* were also detected in fermenting plum mashes. *Metschnikowia* sp. M1, *H. uvarum* H1 and H2 strains were detected in all samples, irrespective of the tested variety and year. Investigation of the impact of individual yeast strains on the production of volatile compounds showed the potential possibility of using them as starter cultures.

## 1. Introduction

Plums are currently among the most popular fruits used for fruit brandy production [1]. In Poland, the most famous is traditional homemade “Śliwowica Łącka”, historically associated with the submontane village of Łącko. It is made by spontaneous plum mash fermentation of the Węgierka Zwykła variety and then repeatedly distilled to obtain 70–75% (*v*/*v*) ethanol content.

The Węgierka Zwykła plum variety is valued for its annual high fertility, but it is very susceptible to the plum pox virus (sharka), which causes enormous losses in fruit production [2]. For this reason, in recent years in the Łącko area other popular sharka-resistant plum varieties, such as Węgierka Dąbrowicka, Čačanska Lepotica and Stanley have begun to be cultivated.

Fruits, including plums, are colonized by a wide range of microorganisms whose quantitative and qualitative participation is closely related to their chemical composition [3]. Volatile compounds produced by these microorganisms during fermentation directly affect the sensory quality and organoleptic characteristics of the final product. It was found that their concentration in distillates also depends on fruit origin, the fermentation process itself with substances produced by yeast metabolism or from the degradation of fruit components, as well as on chemical reactions between these compounds during fermentation, distillation and storage [4,5]. Furthermore, the distinctive aroma of plum brandy could come from the inner layer of the peel plums [6], which may vary depending on the variety used.

While the content of aroma components in different plum brandies has been widely examined [4,5,7,8,9,10,11,12], there is limited data about the yeast community present in the plum mash spontaneous fermentation. So far, the only source of knowledge is our previous study [13], which was restricted to the Węgierka Zwykła plum variety. Determination of the effect of plum variety on the yeast composition during fermentation could lead to improved knowledge about the quality of the resulting product. In addition, it could contribute to the development of a new plum brandy product and creation of a starter culture used for its production.

The aim of this study was to characterize yeast ecology during spontaneous fermentation of plum mashes from different varieties commonly cultivated in southern Poland.

## 2. Materials and Methods

### 2.1. Spontaneous Plum Mash Fermentation

Four varieties of plum fruits (*Prunus domestica* L.)—Węgierka Zwykła, Węgierka Dąbrowicka, Čačanska Lepotica, Stanley—from three orchards in the Łącko area (Lesser Poland Voivodeship, Poland), where “Śliwowica Łącka” is produced, were used in this study. Fruits were harvested in September 2012 and 2013 at maturity.

Healthy and undamaged, non-washed plums were aseptically cut into quarters and weighed out in 500 g per 500 mL sterile glass flasks. The fruits were pressed until the juice covered their surface. The flasks were closed with rubber stoppers with fermentation tubes filled with distilled water. For each variety, assays were performed in triplicate. Alcoholic fermentation was conducted for 30 days at 20 °C. The weight losses were measured daily, to monitor the process.

### 2.2. Yeast Enumeration and Isolation

Samples of fermented juices were aseptically collected on different days of fermentation. Serial decimal juices dilutions were made in Ringer solution (sodium chloride 2.25 g L^−1^, anhydrous calcium chloride 0.12 g L^−1^, sodium bicarbonate 0.05 g L^−1^; POCH S.A, Gliwice, Poland). The appropriate dilution was plated in triplicate on Petri dishes and poured with WL (Wallerstein Laboratory) Agar (BIOCORP, Warsaw, Poland) for total yeast isolation or Lysine Medium (Sigma-Aldrich, Saint Louis, MI, USA) for non-*Saccharomyces* yeast isolation. To avoid bacterial growth, 100 mg L^−1^ of chloramphenicol was added to the media.

After the incubation at 28 °C for 72 h (Lysine Medium) or for 5 days (WL Agar) the colonies were enumerated. Colonies differing in their morphology were randomly selected for identification, compared microscopically and streaked on Sabouraud Dextrose with Chloramphenicol LAB-AGAR (BIOCORP, Poland) to obtain pure cultures. From every sampling time (plum variety, day of fermentation and season) at least five colonies were isolated.

### 2.3. DNA Extraction and RAPD-PCR Analysis

Genomic DNA was isolated from pure yeast cultures using the Yeast Genomic Mini AX Spin (A&A Biotechnology, Gdynia, Poland), following the manufacturer’s instructions.

The RAPD-PCR reaction mixture (50 µL) contained 1× One*Taq* Standard Reaction Buffer, 200 µM of each dNTP, 1.25 U of One*Taq* DNA Polymerase (New England Biolabs, Ipswich, MA, USA), 0.2 µM M13 primer (5′-GAG GGT GGC GGT TCT-3′) (oligo.pl, Poland) and 2 µL of extracted genomic DNA. Amplification was performed in a MultiGene Mini thermocycler (Labnet International, Edison, NJ, USA) using the following thermal program: initial denaturation (95 °C for 5 min), 35 cycles (95 °C for 1 min, 36 °C for 1 min, 68 °C for 2 min) and a final polymerization (68 °C for 7 min).

PCR products were separated on 2% (*w*/*v*) agarose (Lab Empire, Rzeszów, Poland) gels in TAE buffer with ethidium bromide (Sigma-Aldrich, Saint Louis, MI, USA) at 100 V for 60 min. The gels were visualized on a UV transilluminator and photographed on the gel documentation system Felix 1010 (Biostep, Burkhardtsdorf, Germany). Band positions were analyzed visually and compared to a molecular weight marker 100–1000 Ladder (A&A Biotechnology, Gdynia, Poland).

### 2.4. Amplification and Sequencing the D1/D2 Domains of the 26S rRNA Gene Region

D1/D2 domains of the 26S rRNA gene region was amplified using PCR with primers NL1 (5′-GCA TAT CAA TAA GCG GAG GAA AAG-3′) and NL4 (5′-GGT CCG TGT TTC AAG ACG G-3′). The PCR reaction mixture (50 µL) contained 1 x Reaction Buffer, 200 µM of each dNTP, 2.5 mM of MgCl_2_, 1.25 U of Supreme NZY*Taq* II DNA Polymerase (NZYTech, Lisboa, Portugal), 0.2 µM of each primer (Genomed, Warsaw, Poland) and 2 µL of genomic DNA. The temperature program consisted of initial denaturation (95 °C for 15 min), 35 cycles (94 °C for 30 s, 54 °C for 30 s, 72 °C for 1 min) and final polymerization (72 °C for 7 min).

The PCR products were purified using the Clean-up AX (A&A Biotechnology, Poland) following the manufacturer’s instruction and submitted for sequencing to Macrogen Inc. (Amsterdam, the Netherlands). Species identification was carried out by comparing obtained sequences with those available in the GenBank NCBI database at http://www.ncbi.nlm.nih.gov/BLAST/. An identity threshold was considered of at least 99% [14]. Sequences were deposited in the GenBank NCBI database with the accession numbers: MN464117-MN464145.

### 2.5. Production of Volatile Components by Identified Yeast Strains (SPME-GC-TOFMS)

Isolated and identified yeast strains growing over-night (Sabouraud Dextrose Broth;BIOCORP, Warsaw, Poland) were centrifuged (735 g), resuspended in Ringer’s solution and 6 log CFU mL^−1^ were inoculated into YNB solution (Yeast Nitrogen Base; Sigma-Aldrich, Saint Louis, MI, USA) with 0.55% of sucrose, 0.25% of glucose and 0.2% of fructose as a carbon source (average ratio of fermenting sugars in plum mashes). After 10 days of incubation (25 °C), the samples were centrifuged (735 g), and the supernatants were analyzed by SPME-GC-MSTOF. Determination of the volatiles was carried out according to the method described by Zdaniewicz et al. [15]. Compounds were identified using mass spectral libraries and Linear Retention Indices, calculated from a series of n-alkanes from C6 to C30.

The qualitative and quantitative identification of volatile substances (ethyl acetate, isobutyl acetate, isopentyl acetate, ethyl hexanoate, ethyl octanoate, 2-phenylethyl acetate, ethyl decanoate, ethyl dodecanoate, isobutanol, 3-methyl-1-butanol, 2-methyl-1-butanol, 1-hexanol, 1,6-heptadien-4-ol, acetic acid, hexanoic acid, octanoic acid, decanoic acid, diethyl acetal; Sigma-Aldrich, Saint Louis, MI, USA) was based on the comparison of retention times and peak surface area read from sample and standard chromatograms. All tests were carried out in triplicate.

### 2.6. Statistical Analysis

A heatmap representation of the full volatiles data set produced by the isolates (28 yeast strains, 18 volatile components, 3 independent replicates), thus performing a hierarchical cluster analysis (HCA), was constructed using SPSS 18.0 (SPSS Inc., Chicago, IL, USA). HCA is an exploratory tool applied to characterize the data set and reveal natural groupings (or clusters) within it, through the representation of a dendrogram (tree diagram) and heatmap. Squared Euclidean distances were used, and Ward’s minimum variance was used as the clustering algorithm.

## 3. Results

### 3.1. Yeast Population Changes during Spontaneous Fermentation

**Figure 1:**
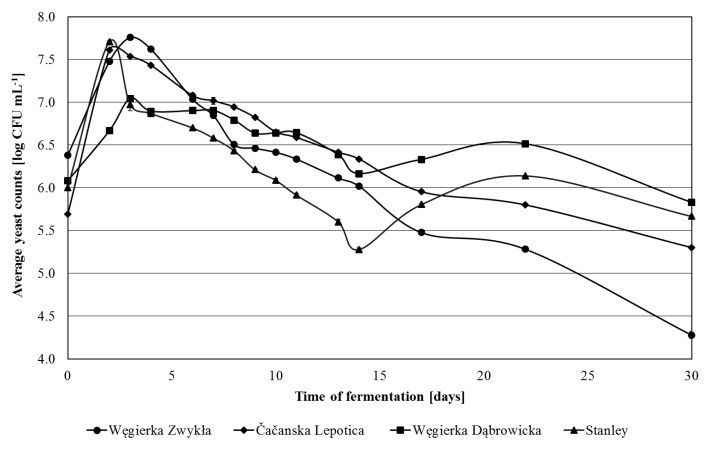
Total counts of yeasts 2012 season in the course of fermentation, *n* = 3, STD < 5%.

In 2012, the largest overall cell count of yeasts in unfermented juice was noted for Węgierka Zwykła variety (Figure 1). For mashes made from Stanley and Čačanska Lepotica fruits, the maximum cell count of yeasts occurred on the 2nd day of fermentation, while in Węgierka Zwykła and Węgierka Dąbrowicka mashes occurred on the 3rd day of fermentation. Next, the yeast cell count began to gradual decrease. On the last day (30th day) the total yeast cell count was ranged from 4.28 log CFU mL^−1^ (Węgierka Zwykła) to 6.83 log CFU mL^−1^ (Węgierka Dąbrowicka).

**Figure 2:**
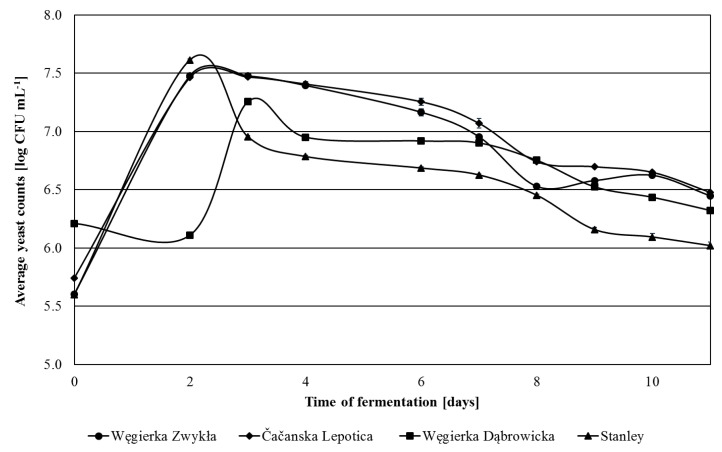
Non-*Saccharomyces* yeasts 2012 season in the course of fermentation, *n* = 3, STD < 5%.

The amount of non-*Saccharomyces* yeasts in fresh mashes mostly did not differ significantly between the varieties and ranged from 5.54 log CFU mL^−1^ (Stanley) to 5.74 log CFU mL^−1^ (Čačanska Lepotica) (Figure 2). In this regard, only Węgierka Dąbrowicka mashes stood out, containing almost four times more non-*Saccharomyces* yeasts, 6.21 log CFU mL^−1^. The maximum level of the discussed yeast group in most mashes was detected on the 2nd day of fermentation. The largest number of non-*Saccharomyces* yeasts were obtained in Stanley mashes, 7.61 log CFU mL^−1^. Węgierka Dąbrowicka mashes stood out again reaching the maximum amount of non-*Saccharomyces* yeasts on the 3rd day, and they were three times lower than in other mashes. In the following days, there was a decrease in the level of non-*Saccharomyces* yeasts, with a sharp decrease in the case of Stanley mashes. The longest high content of non-*Saccharomyces* yeasts was maintained in Čačanska Lepotica mashes. On the 11th day of fermentation, there was still from 6.02 log CFU mL^−1^ (Stanley) to 6.48 log CFU mL^−1^ (Čačanska Lepotica) of non-*Saccharomyces* yeasts.

**Figure 3:**
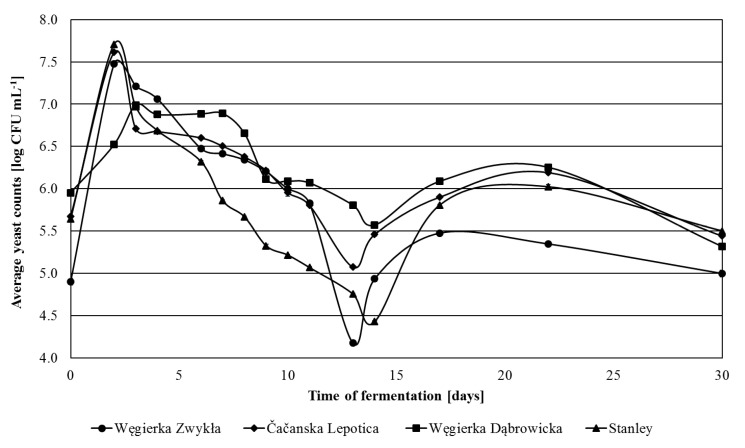
*Kloeckera*/*Hanseniaspora* yeasts 2012 season in the course of fermentation, *n* = 3, STD < 5%.

Additionally, the number of *Kloeckera*/*Hanseniaspora* yeasts was examined. Their colonies on WL agar exhibited the characteristic green color, and in the microscopic image, they were visible as lemon-shaped cells. It was observed that in fresh plum mashes, they represented 96% of yeast microbiota of Čačanska Lepotica fermented mashes, 75% of Węgierka Dąbrowicka, 43% of Stanley and only about 33% of Węgierka Zwykła (Figure 3). Already on the 1st day of fermentation, their number started to grow rapidly and on the 2nd day reached its maximum. The exception was the Węgierka Dąbrowicka mashes, where the maximum number of this yeast group occurred on the 3rd day. The highest level of *Kloeckera*/*Hanseniaspora* yeasts was observed in mashes obtained from Stanley plums, 7.70 log CFU mL^−1^, which accounted 99.2% of all isolated yeasts. In Čačanska Lepotica mashes, their maximum amount was 7.61 log CFU mL^−1^, which was 100% of all isolated yeasts. After the 2nd day of fermentation, the quantity of *Kloeckera*/*Hanseniaspora* yeasts began to diminish. In Čačanska Lepotica mashes, this decline occurred the most rapidly. On the 3rd day of the process their number was 6.71 log CFU mL^−1^, whereas in mashes obtained from the Węgierka Zwykła variety, it was 7.21 log CFU mL^−1^. In the final days of fermentation, there were still detected small amounts of the yeast genus *Kloeckera*/*Hanseniaspora*.

Because of the significant differences in results obtained on Lysine Medium compared to the other media used, in 2013, only WL agar was used. The yeast grew very well, and it was also easier to distinguish one culture from another.

**Figure 4:**
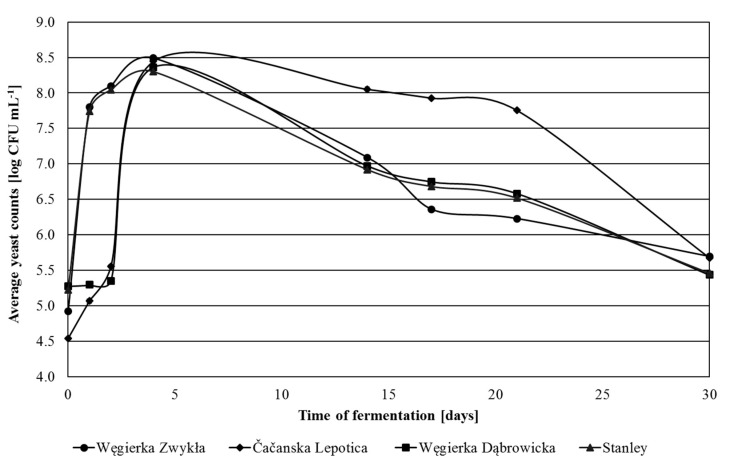
Total counts of yeasts 2013 season in the course of fermentation, *n* = 3, STD < 5%.

In 2013, in the fresh mashes, from 4.54 log CFU mL^−1^ (Čačanska Lepotica) to 5.28 log CFU mL^−1^ (Węgierka Dąbrowicka) of yeast was detected (Figure 4). In Stanley and Węgierka Zwykła mashes on consecutive days there was a rapid increase in the amount of yeast cells, to achieve the highest level on the 4th day of fermentation. In Węgierka Dąbrowicka and Čačanska Lepotica mashes increased yeast growth was recorded after the 2nd day of fermentation. The maximum number of yeasts in fermented plum mashes ranged from 8.30 log CFU mL^−1^ (Stanley) to 8.49 log CFU mL^−1^ (Węgierka Zwykła). After the 4th day, a gradual decrease in the amount of yeasts in all fermenting mashes was noted. The number of yeasts decreased the slowest in Čačanska Lepotica mashes. On the 22nd day, there was still 7.76 log CFU mL^−1^. At the end of the process (30th day) in fermented mashes, there occurred more than 5.43 log CFU mL^−1^ (Węgierka Dąbrowicka) to 5.70 log CFU mL^−1^ (Węgierka Zwykła) yeast cells.

### 3.2. Yeast Identification

170 (in 2012) and 92 (in 2013) pure yeast cultures were isolated from various stages of fermented mashes from four plum varieties.

**Figure 5:**
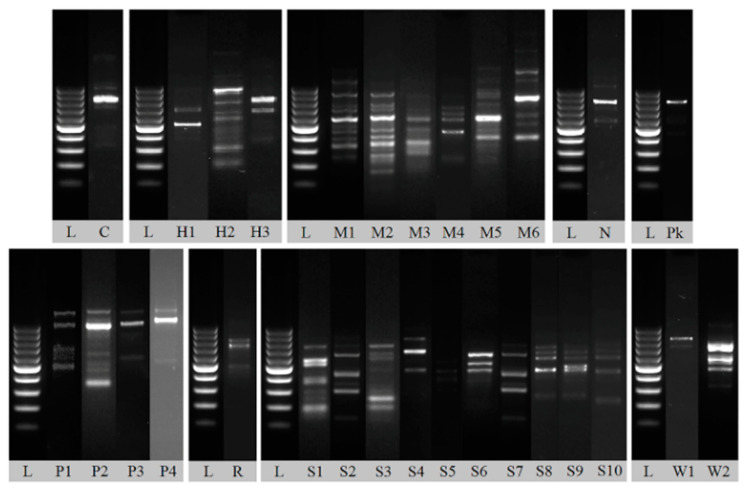
Representative random amplification of polymorphic DNA (RAPD) patterns obtained with primer M13. Lane C: *Candida sake* strain, H1–H3: *Hanseniaspora uvarum* strains, M1–M6: *Metschnikowia* sp. strains, N: *Nakazawaea ernobii* strain, Pk: *Pichia kluyveri* strain, P1–P4: *Pichia kudriavzevii* strains, R: *Rhodotorula mucilaginosa* strain, S1–S10: *Saccharomyces cerevisiae* strains, L: 100–1000 bp DNA Ladder.

Isolates were typed by RAPD-PCR in order to characterize the identical strain and to reduce the number of samples taken for further analysis. All isolates were classified into groups characterized by distinct electrophoretic patterns (Figure 5). Difference even in one band caused the isolate to be included in a separate group.

Representatives of RAPD patterns, one from each group, resulting in 29 isolates, were identified by sequencing the D1/D2 domains of the 26S rRNA gene region (Table 1).

In 2012, the majority of the identified yeast isolates belonged to the *Saccharomyces cerevisiae* species. It was also the most diverse group at the strain level—10 different strains and different RAPD patterns. The other isolates belonged to the: *Metschnikowia* sp. (5 strains), *Pichia kudriavzevii* (4 strains), *Hanseniaspora uvarum* (3 strains), *Wickerhamomyces anomalus* (2 strains), *Candida sake* (1 strain), *Pichia kluyveri* (1 strain), *Nakazawaea ernobii* (1 strain) and *Rhodotorula mucilaginosa* (1 strain).

In 2013, the most identified strains belonged to the *Metschnikowia* sp. (6 strains). *S. cerevisiae* species (5 different strains), *H. uvarum* (3 strains), *P. kudriavzevii* (1 strain) and *W. anomalus* (1 strain) were also identified.

### 3.3. Biodiversity of Yeasts during Fermentation

Analyses showed that in 2012, *Metschnikowia* sp. M1, *H. uvarum* H1, H2, H3 strains were present in fermented mashes of all examined plum varieties.

In Węgierka Zwykła mashes (Table 2), up to the 10th day of fermentation, *Hanseniaspora* strains dominated. Starting from the 6th day of fermentation, in fermented mashes *S. cerevisiae* strains were detected. Węgierka Zwykła mashes revealed the presence of four strains (S1, S2, S3, S5) of the above-mentioned species. With the *P. kudriavzevii* strains, they finished the fermentation process.

In Čačanska Lepotica mashes (Table 3), on the first 3 days of fermentation *Hanseniaspora* strains dominated (similar to Węgierka Zwykła mashes). For the first time, *S. cerevisiae* strains were detected in unfermented mashes but in a small amount. As in the case of Węgierka Zwykła mashes, they began to dominate in mashes on the 6th day, but they were represented by other strains, i.e., S6, S7 and S2. From the 10th day of fermentation in mashes, there were also relatively large amounts of *S. cerevisiae* S1 culture. It remained until the end of fermentation, accounting for 64% of the total yeast microbiota.

In Stanley plum mashes (Table 4), as in the two previously described cases, *Hanseniaspora* sp. was prevalent, up to the 7th day of fermentation. Again, a *S. cerevisiae* strain (S6) was detected relatively early (on the 2nd day), but its domination started from the 7th day. By the end of fermentation, a large amount of strains S2 and S7 was reported.

Composition of the yeast population of Węgierka Dąbrowicka mashes (Table 5) showed slight differences. A relatively large amount of *Metschnikowia* spp. cells occurred already on fruits. The same happened in the fermenting mashes, where even on the 4th day of fermentation representatives of this species were detected. At the same time, presence of *Hanseniaspora* yeasts was reduced. *S. cerevisiae* strains were present in mashes from the 2nd day of fermentation, but their dominance took place from the 11th day.

In 2013, fermenting plum mashes of examined varieties contained notably fewer yeast isolates than in 2012. Again, in all attempts *Metschnikowia* sp. M1 and *H. uvarum* H1, H2 strains occurred. In most attempts *S. cerevisiae* S1 and S2 strains were also present.

In 2013, Węgierka Zwykła plums (Table 2) were dominated by *H. uvarum* H2 strain. Only in this plum variety, in unfermented juice, were *S. cerevisiae* strains present. S2 strain was detected from the beginning until the end of fermentation (on the 30th day) and predominated throughout the whole fermentation process. The non-*Saccharomyces* strains were found only until the 4th day of the process.

In Čačanska Lepotica mashes (Table 3), representatives of *Metschnikowia* sp. M1 and *H. uvarum,* H2 were present in relatively large quantities, through almost the entire process. The only detected *S. cerevisiae* strain, S1, appeared in fermenting mashes on the 7th day of fermentation, to dominate from the 14th day.

Similar to Čačanska Lepotica, also in Stanley mashes (Table 4) were non-*Saccharomyces* yeasts present during the entire process of spontaneous fermentation. *H. uvarum* H2 strain prevailed. In the following days, its amount was still relatively high, representing 16 to 50% of the yeast population. *S. cerevisiae* strains appeared on the 2nd day of fermentation (strain S1) and began to prevail on the 4th day of fermentation. S1, S2 and S7 strains were detected in the largest quantities.

The composition of yeast microbiota in mashes obtained from Węgierka Dąbrowicka plums (Table 5) resembled those from Čačanska Lepotica plums. The first four days were dominated by *H. uvarum* H2 and *Metschnikowia* sp. M1 cultures. Their high participation continued until the end of fermentation process. *S. cerevisiae* strains were observed only from the 7th day. As in the other mashes from 2013, among *S. cerevisiae* there occurred primarily S1 and S2 strains.

### 3.4. Production of Volatile Components by Identified Yeast Strains

**Figure 6:**
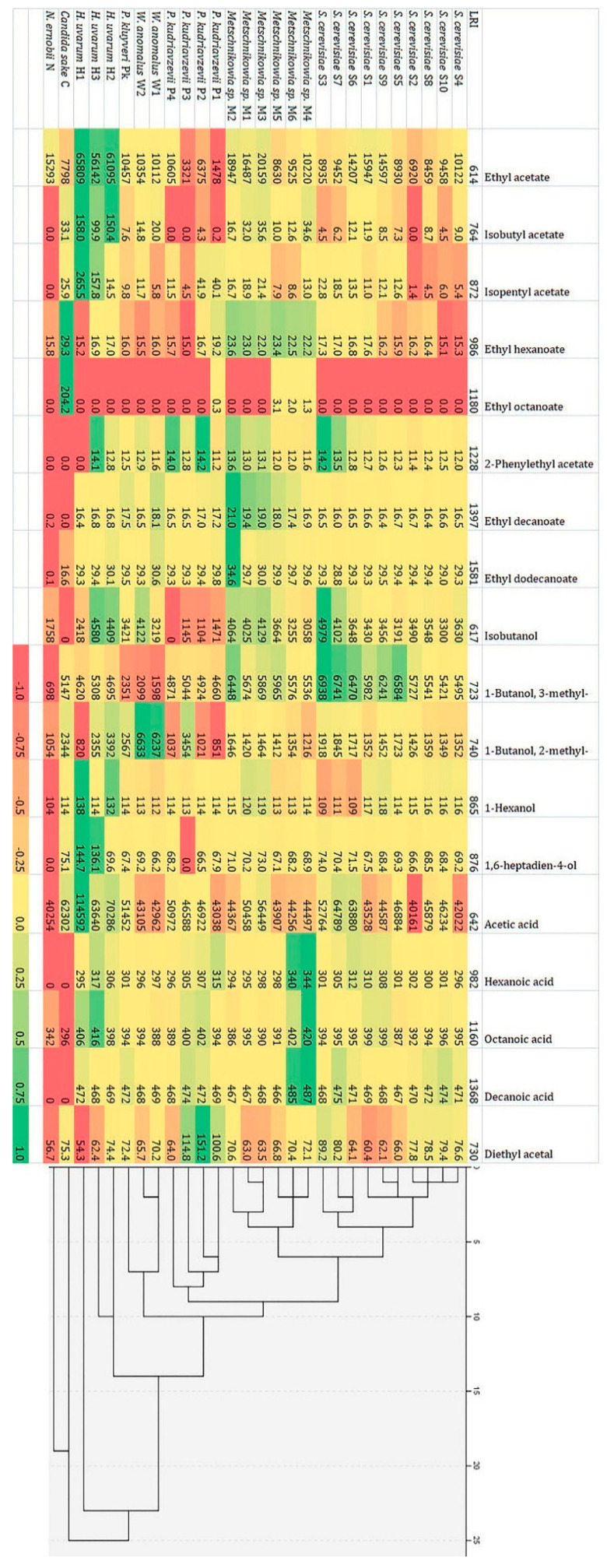
A heat map and cluster analysis results of 18 volatile components [μg L^−1^] produced by yeast strains isolated from various fermenting plum mashes of analyzed varieties (LRI—linear retention time).

Analysis of the main volatile components produced by the identified yeast strains enabled categorization (Figure 6), which was consistent with their species identification. The strain of non-fermenting yeast, *Rh. mucilaginosa,* was not included.

The results showed that strains of *Metschnikowia* sp. are able to produce the widest range of volatile compounds, in contrast to *N. ernobii* and *C. sake*. Two subgroups were found among *Metschnikowia* sp. isolates. First (M4, M5 and M6 strains) formed higher amounts of ethyl octanoate and fatty acids (hexanoic, octanoic, decanoic), second (M1, M2, M3 strains) were characterized by higher production of acetates, ethyl decanoate and isobutanol. The largest concentrations of analyzed volatiles (especially ethyl acetate, isobutyl acetate and isopentyl acetate as well as acetic acid) were produced by *H. uvarum* strains. *W. anomalus* and *P. kudriavzevii* strains were distinguished by the production of high concentrations of 2-methyl-1-butanol and diethyl acetal, respectively. All of the *S. cerevisiae* strains were characterized by the similarity in profile of volatile compounds produced, showing a high ability to form higher alcohols and organic acids production. Based on the ability to synthesize the analyzed volatile components, *S. cerevisiae* isolates were divided into three groups: the first (S2, S4, S8, S10) characterized by the average amount of analyzed components, the second (S1, S5, S9) forming larger amounts of ethyl acetate and 3-methyl-1-butanol, and the third (S3, S6 and S7) producing relatively large amounts of isopentyl acetate, isobutanol, both amyl alcohols and acetic acid.

## 4. Discussion

Quantitative analyses performed for different varieties of plum fruits were generally in agreement with data from previously reported studies of Węgierka Zwykła plum mashes [13] and grape must spontaneous fermentation [16,17,18]. The quantity did not differ significantly between the analyzed varieties.

Non-*Saccharomyces* yeasts are commonly present at the surface of fruits and consequently constitute one of the largest yeast populations during the early stages of alcoholic fermentation. As we expected, in all samples, they prevailed within the first 11 days of the process. A couple of times, it was observed that the number of microorganisms classified as non-*Saccharomyces* yeasts exceeded the number of the total yeast microbiota. Application of different media could affect the misstatement of results. The number of non-*Saccharomyces* yeasts was determined using Lysine agar. These yeasts in contrast to *Saccharomyces* spp. are able to metabolize lysine as the sole nitrogen source. Probably more favorable composition of lysine medium reduced the impact of competition from the yeast *S. cerevisiae*, which allowed non-*Saccharomyces* yeasts to achieve higher numbers than in the WL agar.

During spontaneous fermentation, the largest group of non-*Saccharomyces* yeasts were those belonging to the genus *Kloeckera*/*Hanseniaspora* and usually consists of 50% to 75% of the isolates [19]. Ribereau-Gayon et al. [18] stated that in some cases they can reach even 99%, which is in agreement with our results. Proliferation of *Kloeckera*/*Hanseniaspora* yeasts is an important factor which should be monitored during fermentation because of their potential for rapid depletion of nutrients from the medium and significant contribution to the development of sensory qualities by producing components such as glycerol, esters or acetoin. The scale of synthesis of these compounds varies among species and individual characteristics of the strain [20]. It is assumed that they constitute a risk factor in grape fermentation when their number in an advanced stage of fermentation reaches more than 10% of the total yeast microbiota. In all of the analyzed plum mashes, *Kloeckera*/*Hanseniaspora* yeasts exceeded the limit, but the lowest numbers were observed for the Węgierka Zwykła variety. According to this, it can be supposed that the spontaneous fermentation of mashes obtained from Čačanska Lepotica, Stanley and Węgierka Dąbrowicka plum varieties requires more control. Probably sorbitol, which forms part of plums, had a protective effect on cell walls of non-*Saccharomyces* yeasts, hence their greater participation in plum fermentation [13,21].

The number, composition and diversity of strains in the fermentation process is affected by many factors including variety, chemical composition, maturity stage and condition of fruits, as well as the climatic conditions and agricultural practice in orchards [22,23]. Fruits used in the experiments were obtained from the same orchards, in the same period of harvest and were subjected to the same agricultural technology treatment. For this reason, the most important factor differentiating the quantitative and qualitative profiles of microbiota in examined mashes is the plums variety, which indirectly affects the chemical composition of fruits.

Satora et al. [24] showed that depending on the variety, mashes are characterized by different physicochemical parameters. Concentrations of total and reducing sugars as well as mashes acidity are quite similar among the varieties and seasons. The amounts of free amino nitrogen are varied, but as in the case of other parameters, there is no clear trend correlated with the fruit variety used or the growing season.

In 2013 the number of yeasts during fermentation was approximately ten times lower than in the 2012 season. Moreover, yeast biodiversity during fermentation in the 2013 season was noticeably reduced. Probably this was due to the lower initial number of microorganisms, which could be caused by the different weather conditions during each year. Our other studies investigating the yeast microbiota during spontaneous grape must fermentation [25] conducted in the same seasons (2012 and 2013) confirm that the 2013 year was less favorable for the growth of microorganisms on fruits. It has been shown that yeast amount on the fruit surface declined in the warm season with lack of rainfall [26]. According to Statistical Yearbook of Agriculture [27], the average air temperatures in 2013 in Poland were similar to those in 2012, but there was more rainfall in the summer. In addition, the average cloudiness in 2013 was higher than in 2012, and there were more days of frost.

Qualitative yeast composition during plum mash fermentation showed sequential development of yeasts. It is widely known that oxidative, weakly fermentative or fermentative ascomycetous species such as *Kloeckera*/*Hanseniaspora*, *Metschnikowia*, *Pichia* and *Candida* are predominant in fresh musts and the first stages of spontaneous grape fermentation [21,28,29,30], while *Saccharomyces* spp. end the fermentation process [31]. The most frequently identified non-*Saccharomyces* yeasts belong to *H. uvarum* strains, to a lesser extent to *Metschnikowia* spp. They were present and predominant in all analyzed mashes in consecutive years in the initial stage of fermentation, while *S. cerevisiae* strains were dominant in the middle and final stages.

According to our previous study of Tuszyński and Satora [32], the Węgierka Zwykła plum fruits are colonized mainly by the yeast-like fungi of the genus *Aureobasidium* and *Kloeckera apiculata* yeasts which constitute over 80% of the fungal microbiota. Moreover, Satora and Tuszyński [13] found that Węgierka Zwykła plum fermentation is begun by *K. apiculata* and *Candida pulcherrima* species—anamorphic forms of *H. uvarum* and *M. pulcherrima*. Our research confirms the presence of *H. uvarum* strains. Due to the limitation of the identification method used, we were unable to identify *Metschnikowia* yeast at species level. It is related with high intragenomic diversity of D1/D2 domains of the 26 rRNA of *Metschnikowia* spp. [33,34].

We have managed to isolate *P. kudriavzevii* strains (*Candida krusei* anamorph). It was previously isolated in different parts of the world from grape musts and early stages of fermentation [35,36,37,38], as well as the surface of other fruits [39,40]. In European winemaking, *P. kudriavzevii* is considered one of the non-*Saccharomyces* yeast species that initiate the fermentation process [41]. Vadkertiová et al. [42] found that *P. kudriavzevii*, next to *H. uvarum*, *H. guilliermondii*, *P. kluyveri* and *W. anomalus* is one of the yeast species commonly isolated from plum fruits in southwest Slovakia. In our research, four different *P. kudriavzevii* strains were detected at various stages of fermentation, mainly in the 2012 season. They can be very valuable in the wine industry due to the potential ability of malic acid degradation [43].

We detected through the entire fermentation process strains of *Metschnikowia* sp. M1 in Čačanska Lepotica mashes (2013), *H. uvarum* H2 in Węgierka Dąbrowicka mashes (2013) and *S. cerevisiae* S2 in Węgierka Zwykła mashes (2013). *H. uvarum* H2 strain was also detected in Stanley mashes (2013) at almost all stages of fermentation. During spontaneous fermentation of plum mashes, ethanol concentration is not very high (Figure S1). At the end of process, its concentration in these attempts was 5.03% (v/v) in Čačanska Lepotica mashes, 5.52% (*v*/*v*) in Węgierka Dąbrowicka mashes, 5.83% (v/v) in Węgierka Zwykła mashes and 7.16% (*v*/*v*) in Stanley mashes (determined by pycnometric method) [unpublished data]. Our results are in agreement with the literature data, which indicate that non-*Saccharomyces* strains could be found during the entire process of spontaneous wine fermentation [44,45]. Additionally, some strains of *Metschnikowia* spp. and *H. uvarum* show ethanol tolerance to respectively 5% (*v*/*v*) and 7% (*v*/*v*) [46]. Already mentioned sorbitol could also increase their ethanol tolerance [13]. Furthermore, the dominance of individual strains in mashes of different plum varieties in 2013 could be related to the decline in the abundance and diversity of yeast microbiota in that season, which caused decreased competition and better availability of carbon source and nutrients.

Participation of non-*Saccharomyces* yeast in the later stages of the fermentation process can contribute to the improvement of the complexity of the flavor of the final product. It has been reported that the selective use of fructose by certain *Hanseniaspora* spp. improves the utilization of sugars by the *Saccharomyces* spp. by reducing the risk of occurrence of residual sugars from the fermentation, especially fructose [47]. In addition, it was shown that co-fermentation of *H. uvarum* with *S. cerevisiae* strains can produce wines with acceptable balance and volatile and non-volatile compounds and sensory scores [48]. In grape fermentation, co-fermentation of some strains of *M. pulcherrima* and *S. cerevisiae* was also performed. Wines obtained in this way were characterized by higher content of aromatic compounds compared to mono-culture fermentation [49]. Moreover, in sensory tests, these wines were preferred, in contrast to those obtained only by *M. pulcherrima* strains, which were overly estery [3]. The test of the production of volatile compounds by individual yeast strains confirmed that non-*Saccharomyces* yeasts are able to enhance the sensory properties of the product. Moreover, use of *Metschnikowia* sp. M1, H. uvarum H1 and H2 (detected in all of the analyzed samples) as a starter culture could enrich fermented beverages with esters and could provide characteristic features of the product.

Twice (in 2012 and 2013) we isolated *S. cerevisiae* strains from unfermented plum mashes of Čačanska Lepotica and Węgierka Zwykła varieties. In addition, strain S2 isolated from the Węgierka Zwykła mashes could be maintained throughout the fermentation period. It is in agreement with the previous ecological studies on grape must, which showed that they exist in unfermented juice, but their numbers reach very low levels, usually below the detection limit [50].

Regardless of the season and used plum variety, occurrence of common strains in mashes was observed. It confirms that the geographic location and microclimatological conditions—in our case a submontane climate—have a large impact on microbiota composition during fermentation [18,22,51,52]. We did not observe, like Raspor et al. [51], that particular yeast species show preferences for certain varieties. This may indicate that the plum microbiota is associated with the orchard and the specified area. This may also explain why individual strains were found in consecutive years. Similar conclusions have been reached in the case of grape fermentation [53,54,55].

## 5. Conclusions

The results of the present study provided an overview of the yeast community of spontaneous mash fermentation of different plum varieties. We proved that plum mash spontaneous fermentation is similar to grape must fermentation. The apiculate yeast *H. uvarum* and the non-*Saccharomyces* yeasts were predominant at the early stages of fermentation, while *S. cerevisiae* strains take over the process from around the 10th day. The variety of fruit did not significantly affect the yeast ecology during fermentation. The weather conditions during each season have much more influence on the yeast community. This indicates the possibility of using each of the varieties for the production of slivovitz. However, due to the high content of non-*Saccharomyces* yeasts, the plum fermentation process should be monitored. In addition, we were able to isolate three strains (*Metschnikowia* sp. M1, *H. uvarum* H1 and H2) which were detected in all attempts, in different amounts, as well as three strains (*Metschnikowia* sp. M1, *H. uvarum* H2 and *S. cerevisiae* S2) that could colonize certain mashes at each stage of fermentation. Considering the volatile compounds production by these strains, it is worth determining whether their use as a starter cultures for plum brandy production results in a characteristic sensory profile of the product. It may turn out that plum mash fermentation could be more predictable while simultaneously maintaining the traditional, unique characteristics of the final product.

## Figures and Tables

**Table 1:** Yeast identification by the 26S rRNA D1/D2 domains sequencing.

Strain Symbol	Identification	% Identity	Query Length [bp]	Query Cover	GenBank Accession No.	Number of Isolates
C	*Candida sake*	99.82%	561	100%	MN464117	2
H1	*Hanseniaspora uvarum*	100.00%	569	100%	MN464118	20
H2	*Hanseniaspora uvarum*	100.00%	566	100%	MN464119	43
H3	*Hanseniaspora uvarum*	100.00%	556	100%	MN464120	16
M1	*Metschnikowia* sp.	99.59%	490	100%	MN464121	33
M2	*Metschnikowia* sp.	99.59%	492	100%	MN464122	6
M3	*Metschnikowia* sp.	99.39%	495	100%	MN464123	3
M4	*Metschnikowia* sp.	99.80%	507	98%	MN464124	6
M5	*Metschnikowia* sp.	99.39%	493	100%	MN464125	6
M6	*Metschnikowia* sp.	99.39%	494	100%	MN464126	3
N	*Nakazawaea ernobii*	99.29%	561	99%	MN464127	1
Pk	*Pichia kluyveri*	100.00%	567	100%	MN464128	3
P1	*Pichia kudriavzevii*	99.82%	562	100%	MN464129	1
P2	*Pichia kudriavzevii*	100.00%	556	100%	MN464130	4
P3	*Pichia kudriavzevii*	100.00%	470	100%	MN464131	6
P4	*Pichia kudriavzevii*	100.00%	559	99%	MN464132	7
R	*Rhodotorula mucilaginosa*	99.82%	562	100%	MN464133	2
S1	*Saccharomyces cerevisiae*	100.00%	569	100%	MN464134	19
S2	*Saccharomyces cerevisiae*	100.00%	572	99%	MN464135	30
S3	*Saccharomyces cerevisiae*	100.00%	567	100%	MN464136	9
S4	*Saccharomyces cerevisiae*	100.00%	570	100%	MN464137	1
S5	*Saccharomyces cerevisiae*	100.00%	567	100%	MN464138	2
S6	*Saccharomyces cerevisiae*	99.63%	542	100%	MN464139	4
S7	*Saccharomyces cerevisiae*	99.65%	565	100%	MN464140	17
S8	*Saccharomyces cerevisiae*	99.82%	571	100%	MN464141	4
S9	*Saccharomyces cerevisiae*	100.00%	573	99%	MN464142	5
S10	*Saccharomyces cerevisiae*	99.47%	565	100%	MN464143	2
W1	*Wickerhamomyces anomalus*	100.00%	558	100%	MN464144	5
W2	*Wickerhamomyces anomalus*	100.00%	562	100%	MN464145	2

**Table 2:** Yeast strains (%) isolated from different stages of Węgierka Zwykła plum mash fermentation (2012 and 2013 seasons).

Strain	2012							2013						
	Sampling Day							Sampling Day						
	0	2	3	6	10	22	30	0	1	2	4	7	14	30
*Hanseniaspora uvarum* H1	21							17						
*Hanseniaspora uvarum* H2				57	40			50	29	14				
*Hanseniaspora uvarum* H3		43	83		40				14	10				
*Metschnikowia* sp. M1	32							17						
*Metschnikowia* sp. M5											5			
*Pichia kudriavzevii* P1		14												
*Pichia kudriavzevii* P2						80								
*Pichia kudriavzevii* P3							20							
*Pichia kudriavzevii* P4		43	17								15			
*Saccharomyces cerevisiae* S1					20		60							
*Saccharomyces cerevisiae* S2				14		20		16	57	43	20	65	60	51
*Saccharomyces cerevisiae* S3				29						33	32	15	20	
*Saccharomyces cerevisiae* S5							20							
*Saccharomyces cerevisiae* S7											28	20	20	49
*Wickerhamomyces anomalus* W1	47													

**Table 3:** Yeast strains (%) isolated from different stages of Čačanska Lepotica plum mash fermentation (2012 and 2013 seasons).

Strain	2012							2013						
	Sampling Day							Sampling Day						
	0	2	3	6	10	22	30	0	1	2	4	7	14	30
*Candida sake* C	11													
*Hanseniaspora uvarum* H1	39							15						
*Hanseniaspora uvarum* H2		50	80	43	25			54	33	38	33	20	17	
*Hanseniaspora uvarum* H3					8	25								
*Metschnikowia* sp. M1	39		20					31	67	54	50	20	16	20
*Pichia kluyveri* Pk		50												
*Pichia kudriavzevii* P2	6													
*Pichia kudriavzevii* P3						25	18			8	17	20		
*Rhodotorula mucilaginosa* R						12								
*Saccharomyces cerevisiae* S1	5				42	25	64					40	51	80
*Saccharomyces cerevisiae* S2				29										
*Saccharomyces cerevisiae* S5							9							
*Saccharomyces cerevisiae* S6				14										
*Saccharomyces cerevisiae* S7				14	17									
*Saccharomyces cerevisiae* S8					8									
*Saccharomyces cerevisiae* S9							9							
*Wickerhamomyces anomalus* W2						13							16	

**Table 4:** Yeast strains (%) isolated from different stages of Stanley plum mash fermentation (2012 and 2013 seasons).

Strain	2012								2013						
	Sampling Day								Sampling Day						
	0	2	3	4	7	11	23	30	0	1	2	4	7	14	30
*Hanseniaspora uvarum* H1	52			40					16						
*Hanseniaspora uvarum* H2	5	75							25	50		49	30	32	16
*Hanseniaspora uvarum* H3			100	40	11				25		34				
*Metschnikowia* sp. M1	39								18						
*Metschnikowia* sp. M3											16				
*Metschnikowia* sp. M6									16	20					
*Pichia kudriavzevii* P2	4			29	10										
*Pichia kudriavzevii* P3							17	50							
*Saccharomyces cerevisiae* S1										30	16	17	14		
*Saccharomyces cerevisiae* S2					33	75	33	17				17	28	51	67
*Saccharomyces cerevisiae* S3											34				
*Saccharomyces cerevisiae* S6		25										17	14		
*Saccharomyces cerevisiae* S7					44	25	17	33					14	17	17
*Saccharomyces cerevisiae* S9					11		33								
*Saccharomyces cerevisiae* S10				20											

**Table 5:** Yeast strains (%) isolated from different stages of Węgierka Dąbrowicka plum mash fermentation (2012 and 2013 seasons).

Strain	2012								2013						
	Sampling Day								Sampling Day						
	0	2	3	4	7	11	23	30	0	1	2	4	7	14	30
*Hanseniaspora uvarum* H1	20		20						20						
*Hanseniaspora uvarum* H2		50	40		37				20	40	45	50	34	28	32
*Hanseniaspora uvarum* H3			20							20	10				
*Metschnikowia* sp. M1	25								38	40	45	50	16	14	
*Metschnikowia* sp. M2	20	25							10						
*Metschnikowia* sp. M3	5		20												
*Metschnikowia* sp. M4	5			40					12						
*Metschnikowia* sp. M5	20			10											
*Nakazawaea ernobii* N				10											
*Rhodothorula mucilaginosa* R1	5														
*Saccharomyces cerevisiae* S1				10									17	14	34
*Saccharomyces cerevisiae* S2							100						33	42	
*Saccharomyces cerevisiae* S3															34
*Saccharomyces cerevisiae* S4					13										
*Saccharomyces cerevisiae* S7				30	24										
*Saccharomyces cerevisiae* S8		25				80		100							
*Saccharomyces cerevisiae* S9						20									
*Saccharomyces cerevisiae* S10					13										
*Wickerhamomyces anomalus* W1					13										

## Supplementary Materials

The following are available online at https://www.mdpi.com/2304-8158/9/8/1054/s1, Figure S1: Ethanol concentration in the course of fermentation (determined by HPLC method).
