# Supplementary figures and images for: Growth Dynamics and Diversity of Yeasts during Spontaneous Plum Mash Fermentation of Different Varieties
Source: Foods. 2020 Aug 4;9(8):1054. doi: 10.3390/foods9081054 (PMC7466356; doi:10.3390/foods9081054)

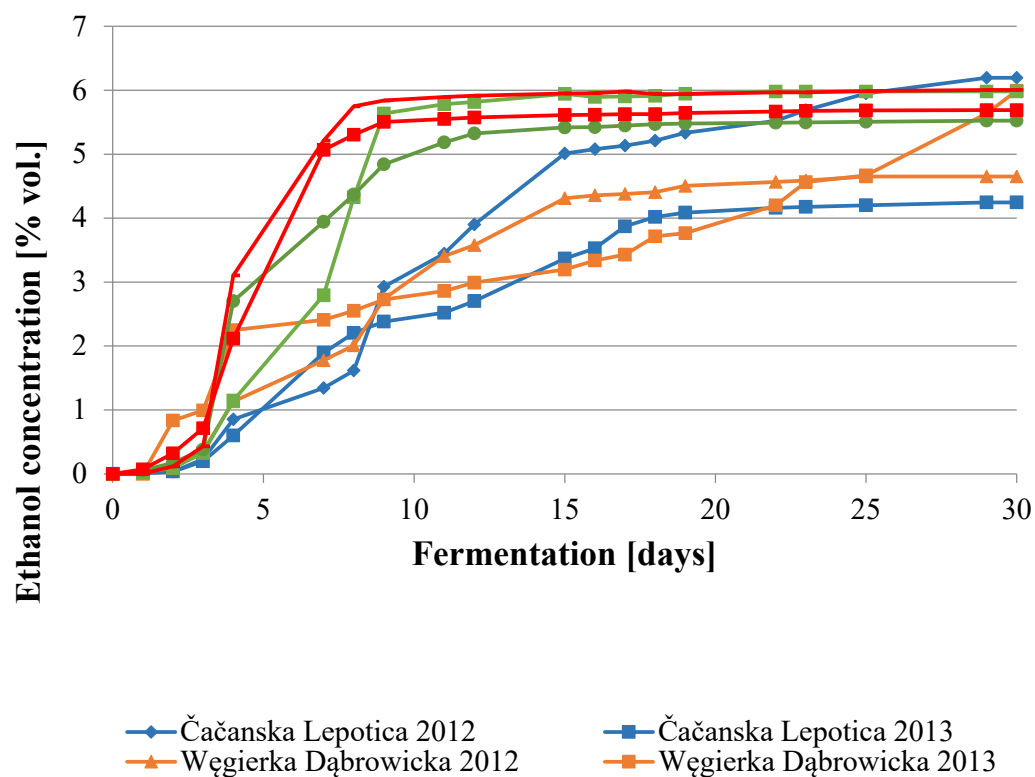

**Figure 1.** Ethanol concentration in the course of fermentation (determined by HPLC method).

Supplement: Supplementary file 1 [file foods-09-01054-s001.pdf]
